# Resilience, stress and injuries in the context of the Brazilian elite rhythmic gymnastics

**DOI:** 10.1371/journal.pone.0210174

**Published:** 2018-12-31

**Authors:** Renan Codonhato, Victor Rubio, Paulo Márcio Pereira Oliveira, Camila Ferezin Resende, Bruna Akawana Martins Rosa, Constanza Pujals, Lenamar Fiorese

**Affiliations:** 1 Physical Education Department, Universidade Estadual de Maringá, Maringá, Paraná, Brazil; 2 Department of Biological and Health Psychology, Universidad Autónoma de Madrid, Madrid, Spain; 3 Physical Therapy Department, Universidade Federal do Sergipe, Lagarto, Sergipe, Brazil; 4 Brazilian Gymnastics Confederation (CBG), Aracajú, Sergipe, Brazil; 5 Psychology Department, Centro Universitário Ingá (UNINGÁ), Maringá, Paraná, Brazil; University of Rome, ITALY

## Abstract

The study had the goal to study the relationship between resilience, stress and injuries in the sport context. Eight female athletes, part of the Rhythmic Gymnastics Brazilian Team along the Olympic Cycle 2015–2016 participated in the study, with a mean age of 20.4±2.5 years. The following instruments were used: RESTQ-76 Sport, CD-RISC 10, documental analysis of physical therapy records, and structured questionnaire. Data was analyzed by descriptive statistics (frequency, mean and standard deviation); Repeated Measured ANOVA with Bonferroni’s post-hoc, Student’s “t” test, Friedman test, Pearson Correlation Coefficient, Cohen’s d, and inductive thematic analysis. We found relatively stable levels of stress and recovery across the season; total recovery levels were higher than stress at all four measured timepoints (p<0.05); All athletes had at least one injury, with a total of 14 injuries; No significant correlations were found between the quantitative scores of resilience, stress and recovery; Training and the sport’s scoring system were the most relevant perceived stressors; athletes presented meta-cognition and a non-positive evaluation (neutral) of stressors; Social support was considered the main psychological factor for the resilience process; such process resulted in improved control and interpretation of emotions; Our hypothesized model proposes that, in the relationship between stress and injuries, resilience acts by optimizing the injury recovery process. It was concluded that resilience plays a role in the process of injury rehabilitation and stress control in elite rhythmic gymnastics’ athletes.

## Introduction

Elite sports can an environment for stressful situations, due to the challenges, difficulties and adversities present in such context. High training loads and high-demand competitions require maximum levels of performance from the athletes, which can cause great physical and mental exhaustion, as well as exposing these athletes to an elevated risk for injury from either exhaustion or direct physical contact [1–4]. Besides these physical variables, the high-stress environment of elite sports can affect injury occurrence through stress, believed to be the main psychological variable related to sport injury [5]. In this sense, psychological resilience can play an essential role in attenuating undesirable effects from the stress process, as resilience is an indicator of the capacity to face and cope with possible adverse situations [6].

The positive relationship between resilience and stress seems to be clear in the qualitative literature [3, 7–9], while the quantitative literature presents direct inverse correlations to stress and stress correlates [10–13], resulting in lower levels of anxiety and higher levels of self-concept, self-esteem, psychological well-being and adaptive coping strategies [14–18]. In this sense, psychological resilience could be a protective factor for injury due to its relationship to stress; however, no studies have investigated the relationship between resilience, stress and injury in elite athletes.

The main mechanisms by which stress might increase injury risk seem to be an increase in muscle tension, higher physical fatigue, reduced flexibility and motor coordination, lower muscular efficacy and peripheral vision disturbances [1, 19, 20]. Injury susceptibility also depends on multiple factors such as internal risk factors (age, gender, body composition, health, physical fitness, anatomy and skill level), external risk factors (opponents, use of protective equipment and environmental factors, such as weather, surface, and maintenance) [21], and the interaction between potentially-stressful situations and athletes’ characteristics, such as neuroticism levels, anxiety levels, coping resources, self-efficacy and history of stressors [1, 2, 22, 23].

Although no study has yet assessed the effects of resilience over injury, it is proposed that positive psychological characteristics, like hardiness and coping, may reduce athletes’ vulnerability to injury, due to their buffering effects [5, 24]. As mentioned above, psychological resilience can also influence stress levels in a positive manner, possibly lowering injury risk indirectly. Another important variable is recovery. Recovery describes a complex process thought to have a regulatory function over stress, as stress levels are detrimental if adequate recovery levels are not reached [25].

Therefore, the present study aims to study the relationship between resilience, stress and injury occurrence in the context of elite sports, specifically analyzing the context of elite rhythmic gymnastics in Brazil and adopting the Grounded Theory of Psychological Resilience and Optimal Sport Performance [3] as the theoretical framework. Our hypothesis is that resilience will play a role in controlling stress levels and aiding in recovery, which will lead to a lower prevalence of injuries and better rehabilitation.

## Materials and methods

### Study characteristic

The present study can be characterized as having mixed methods, for using both quantitative and qualitative approaches, being conducted in a sequential manner, conducting the quantitative part first, followed by the qualitative investigation [26]. This type of study involves and complements the different strong and weak aspects of each method, enriching the overall quality of the investigation.

### Sample

The sample was composed of athletes who were part of the Rhythmic Gymnastics Brazilian Team between July 2015 and August 2016, during the team’s preparation for the 2016 Olympic Games in Rio de Janeiro. They were a total of 08 female athletes with ages between 18 and 26 years, a mean age of 20.4±2.5. All 08 athletes participated in the quantitative part of the investigation; however, for the qualitative portion, one of the athletes could not participate due to a severe injury that kept her away from the team during the qualitative data collection, therefore, only 07 of the 08 athletes answered the structured questionnaire. Although this one athlete was not able to participate in the qualitative analysis, we opted to retain as much as information as possible and so to not exclude her data from the quantitative analysis.

### Instruments

#### Psychological assessments

To identify stress and recovery levels at four different time points in the season the RESTQ-76 Sport (Recovery and Stress Questionnaire for Sports) [27] was used, in its validated version for Portuguese [28]. This instrument is composed of 77 items answered in a 07-point Likert-type scale (from 0 –“Never” to 06 –“Always”). Results are grouped in 10 stress subscales (general stress, emotional stress, social stress, conflicts/pressure, fatigue, lack of energy, physical complaints, disturbed breaks, emotional exhaustion and injuries) and 09 recovery subscales (success, social recovery, physical recovery, general well-being, quality of sleep, being in shape, personal accomplishment, self-efficacy and self-regulation). Total scores are obtained separately for each of the 19 dimensions and range from 0 to 06. Score interpretation were the same for both stress and recovery; therefore, higher scores indicate higher levels of that variable. Cronbach’s alpha indicated adequate reliability (α ≥ 0.70) for most variables, ranging from 0.70 to 0.84 for the stress subscales, and from 0.73 to 0.91 for the recovery dimensions, with the exception of “Success” (α = 0.62) and “Physical recovery” (α = 0.47), which were not excluded but should be interpreted with caution.

The 10-Item Connor-Davidson Resilience Scale (CDRISC-10) [29] was used, in its validated version to the general Brazilian context [30], to assess athletes’ resilience levels at two time points: at the beginning of the investigation, about one year before the Olympic games, and at the end of the investigation, days before this major competition. This scale is composed of 10 items in a 05-point Likert-type scale. The result is obtained by the sum of all items, generating a single-factor result ranging from 0 to 40 points, where higher scores will indicate higher levels of resilience. This instrument presented Cronbach’s alpha = 0.72

Qualitative assessment was performed through a structured questionnaire with open-answer questions; the questionnaire was developed by the authors to assess both the resilience process and the injury/rehabilitation process.

The Grounded theory of psychological resilience and optimal sport performance [3] was used for the development of the resilience-related questions and considered the following factors: a) competitive, organizational and personal stressors (*e*.*g*. training demands or issues in competitions); b) challenge appraisal and meta-cognitions (*e*.*g*. self-awareness and perceived growth opportunities); c) positive personality (*e*.*g*. optimism toward results); d) motivation (*e*.*g*. personal interests); e) focus (*e*.*g*. being able to avoid distractors); f) perceived social support; g) confidence (*e*.*g*. using past experiences); h) facilitative responses (*e*.*g*. decision making) and i) sport performance (*e*.*g*. desired/attained performances).

This model has been developed with Olympic champions and describes the resiliency process of athletes from their perception and interpretation of an adversity (a and b), considering the psychological factors involved in this process (c to f), to the responses in face of such adversity (h), which can lead to differences in sport performance (i). The authors suggest that these psychological factors may protect athletes from the deleterious effects of stressors by influencing the way they perceive and deal with adversity, leading to facilitative responses.

To design the injury-related questions, the Stress-Injury Model [1] was used as a guide. This model has been consistently used to assess the relationship between injury and psychological factors in sports [5] and suggests that the following aspects should be evaluated when studying these variables: a) stressful situations; b) stress history (*e*.*g*. past experiences with stressful situations); c) personality characteristics (*e*.*g*. perception of self-control and anxiety); d) coping resources (*e*.*g*. mental skills); e) stress response (*e*.*g*. feelings during stressful situations); f) interventions (for rehabilitation and to reduce injury vulnerability); g) injuries (*e*.*g*. frequency, type, duration, rehabilitation process and return to practice). Both models share similarities for assessing individual and situational characteristics and were used complementarily. In addition, the Stress-Injury Model provides information regarding injury-specific information to be gathered.

#### Physical therapy records

Physical therapy registers were used to assess injury history and characteristics throughout the season. Such registers were already kept by the team’s staff and the required data were provided in individual sheets for each athlete’s injury record. Athletes were characterized and recorded as injured when they were withdrawn from their daily training routines, either partially (not performing parts of the training routine) or completely (complete day off from training). Information was collected regarding injury severity (minor, mild and severe) and injury duration, evaluated by the time (in days) that the athlete had to refrain from training and/or competition.

### Procedure

This study is part of a project entitled “Development process of positive psychological variables in the sports context” approved by the State University of Maringá ethics committee under the opinion number 1.823.503. Initially, the National Rhythmic Gymnastics Training Center, located in Aracajú-SE, Brazil, was contacted to obtain authorization for data collection. The study goals and procedures were explained to the staff and the athletes, who agreed to participate. Written informed consent was obtained from the coach, while athletes provided verbal consent.

Data collection started in July 2015 and lasted until August 2016, two days before the team traveled for the 2016 Rio Olympic Games. During this period the team participated in four competitions, representing four time points: 1) the 2015 Pan-American Games, 2) the 2015 World Rhythmic Gymnastics Championship, 3) the 2016 Rhythmic Gymnastics World Cup Series (in Berlin, Germany and Kazan, Russia), and 4) the 2016 Olympic Games.

In order to assess pre-competitive levels of stress and recovery, RESTQ-76 was answered within a week before each competition. To evaluate athlete’s resilience and their resilient development after a year of training for such an important competition, CD-RISC-10 was answered once at the beginning of the season (July 2015) and again at the end (August 2016). Injury data were collected and provided by the team’s staff throughout the investigation period. The qualitative questionnaire was answered prior to the 2016 Olympic Games, allowing athletes to consider the entire training season for their answers.

### Data analysis

For quantitative data analysis, the Shapiro-Wilk normality test was performed as a “first step”, showing normal distribution for the stress, recovery and resilience data. Therefore, descriptive statistics were presented in frequency, mean and standard deviation. Repeated measures ANOVA, with Bonferroni’s Post-hoc was used to compare stress and recovery levels within its subscales for each of the four time points; the paired sample T-test was used to compare changes in resilience from the beginning to the end of the season, as well as differences between total stress and total recovery in each of the four time points. Pearson correlation coefficient was adopted to assess the relationship between variables, and Cohen’s D was used for effect size. Analysis were performed on IBM SPSS v.22, with significance level set for p<0.05.

Qualitative data were analyzed through thematic inductive analysis [31]. According to its recommendations, the questionnaires were read and transcribed digitally to software MS Excel 2010 to be manually analyzed, athletes’ answers were then discussed by two of the authors in order to inductively identify and highlight initial codes (words or phrases related to the content of the question) representing a certain characteristic. These data originated low-order themes, which were then grouped in to high-order themes, and then in to the general dimensions proposed by the model used as reference [3].

## Results

### Quantitative results

#### Stress and recovery

**Fig 1:**
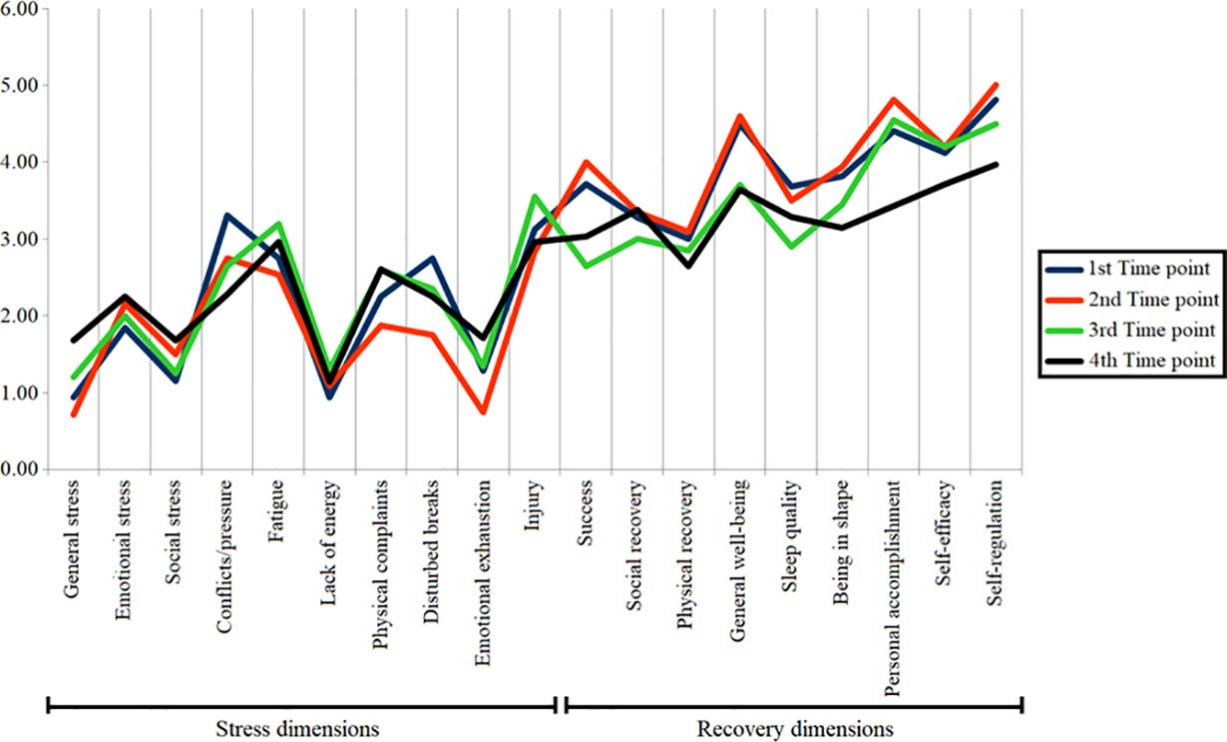
Athletes’ recovery and stress profile throughout their preparation for the 2016 Olympic Games.

The stress and recovery questionnaire was answered four times during this Olympic training cycle, as described in the data collection procedures. No significant differences were found for the scores at any time points, demonstrating that stress and recovery levels were relatively stable throughout the season (Fig 1).

**Fig 2:**
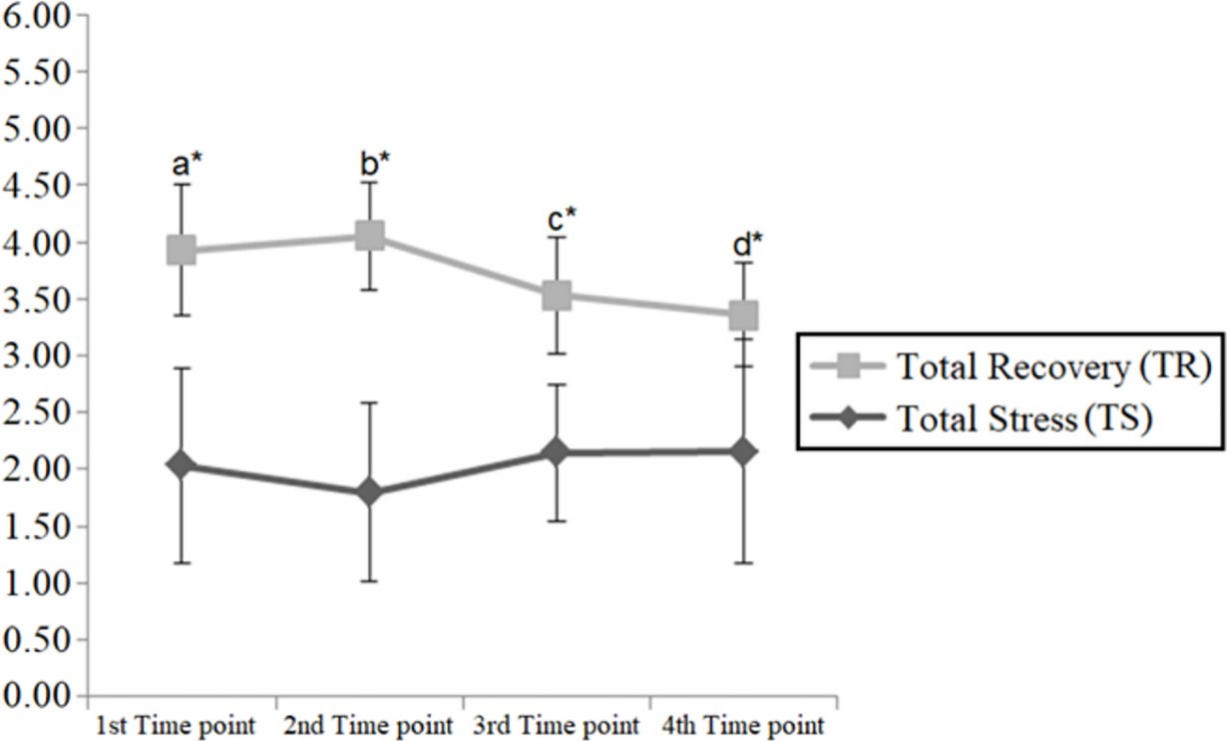
Comparison of athletes’ total stress and total recovery their preparation for the 2016 Olympic Games. *Notes*: TS = Total stress; TR = Total recovery; a = 1^st^ time point diference between TS and TR (t = -4.11, SE = 0.46, p<0.01; d = 2.59); b = 2^nd^ time point difference between TS and TR (t = -5.96, SE = 0.38, p<0.01; d = 3.47); c = 3^rd^ time point difference between TS and TR (t = -3.16, SE = 0.44, p = 0.03; d = 2.47); d = 4^th^ time point difference between TS and TR (t = -2.57, SE = 0.47, p = 0.04; d = 1.56).

By calculating the average of the 10 stress dimensions and the 09 recovery dimensions it is possible to obtain representative values for Total Stress and Total Recovery. The comparison of such variables along the season (Fig 2) has shown that recovery was consistently and significantly higher than stress (p<0.05). However, it is worth noting that the size the difference decreased before the two last competitions, as the athletes were getting near the Olympic Games, presenting a slight increase in the average total stress as well as a slight decrease in total recovery scores.

#### Injury profile

All of the investigated athletes had at least one injury during the investigated period (Table 1). There were 07 (50%) minor injuries, 02 (14.3%) mild injuries and 05 (35.7%) severe injuries, for a total of 14 injuries occurring between July 2015 and July 2016. Among the eight injured athletes, only two (25%) were not restricted from sport-related activities due to injury, but had lower training loads in their daily routines; the other six athletes accounted for a total of 509 days away from sport-related activities as a result of injury. This represents an average of 85 days, approximately three months, away from training and/or competition for each athlete, representing about 46 days of withdrawal per injury suffered. Additionally, from the eight athletes who initially composed the group, three (37.5%) had to be cut from the final team due to severe injuries.

**Table 1:** Athletes’ injury prevalence and characteristics throughout their preparation for the 2016 Olympic Games.

Athletes	Injury severity				Time in rehabilitation^a^
	Minor	Mild	Severe	Total	
A1*	01	01	01	03	147
**A2**	01	-	-	01	33
**A3**	02	-	-	02	0
A4*	-	-	01	01	42
**A5**	01	-	01	02	112
**A6**	-	01	-	01	21
A7*	-	-	02	02	154
**A8**	02	-	-	02	0
**Total (f/%)**	07 (50%)	02 (14.29%)	05 (35.71%)	14 (100%)	509

*Athlete cut off from the Olympic Games due to injury

a = Measured in full-days of withdrawn from training/competition

f = Absolute frequency; % = Relative to the total number of injuries.

#### Resilience

Regarding athletes’ resilience levels (Table 2), the team presented an average score of 29.13±4.09 from the resilience scale at the beginning of the study, and reaching 30.63±4.31 points at the end of this training cycle (p>0.05; d = 0.15), a non-significant low-effect increase. When analyzing each athlete separately, we highlight Athlete 02 with a 30.4% increase in her score, and Athlete 07, with a 15.4% decrease.

**Table 2:** Athletes’ resilience levels at the beginning and the end of an Olympic preparatory year.

Athletes	Resilience		Difference
	First time point	Last time point	
**A1**	26.00	29.00	+11.54%
**A2**	23.00	30.00	+30.43%
**A3**	32.00	34.00	+06.25%
**A4**	34.00	35.00	+02.94%
**A5**	27.00	29.00	+07.41%
**A6**	33.00	35.00	+06.06%
**A7**	26.00	22.00	-15.38%
**A8**	32.00	31.00	-03.12%
**Team average**	29.13±4.09	30.63±4.31	+01.50±0.22

Team average is presented in Mean ± Standard Deviation. Comparison between first and last time points: p>0.05; d = 0.15.

No significant correlations were found between resilience, either at the beginning or end of the season, and total stress or total recovery at any of the four time points. For each specific stress and recovery dimension, initial resilience was correlated with Success (r = -0.72) and Well-being (r = 0.77) at time point 01; Success (r = -0.83) at time point 02; and Social Recovery (r = 0.91) at time point 03; end-season resilience correlated with Success (r = -0.79) at time point 1; and Fatigue (r = 0.82), Physical Complaints (r = 0.98) and Disturbed Breaks (r = 0.72) at time point 02. However, due to the small sample size, high r values and inconsistency through measurements, these correlations were interpreted as spurious.

#### Team’s performance

Although evaluating the team’s performance was not the main goal of the present investigation, it is worth presenting the actual performance achieved by the athletes, which is the main outcome of the entire process observed during this study as well as the final element of the model adopted as theoretical framework. Despite all injuries, the team successfully fulfilled their previously established goals: gold medal for the 2015 Pan-American Games and top-10 in the Olympic Games (9^th^ place).

### Qualitative results

**Fig 3:**
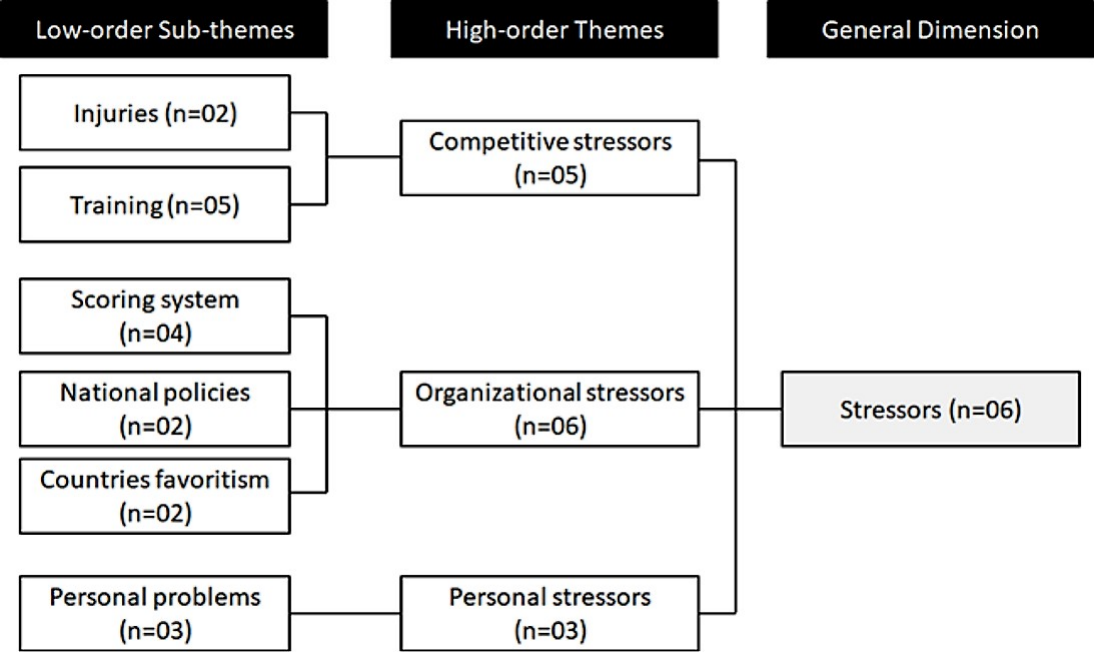
Stressors’ characteristics perceived by the athletes during their training for the 2016 Olympic Games.

**Fig 4:**
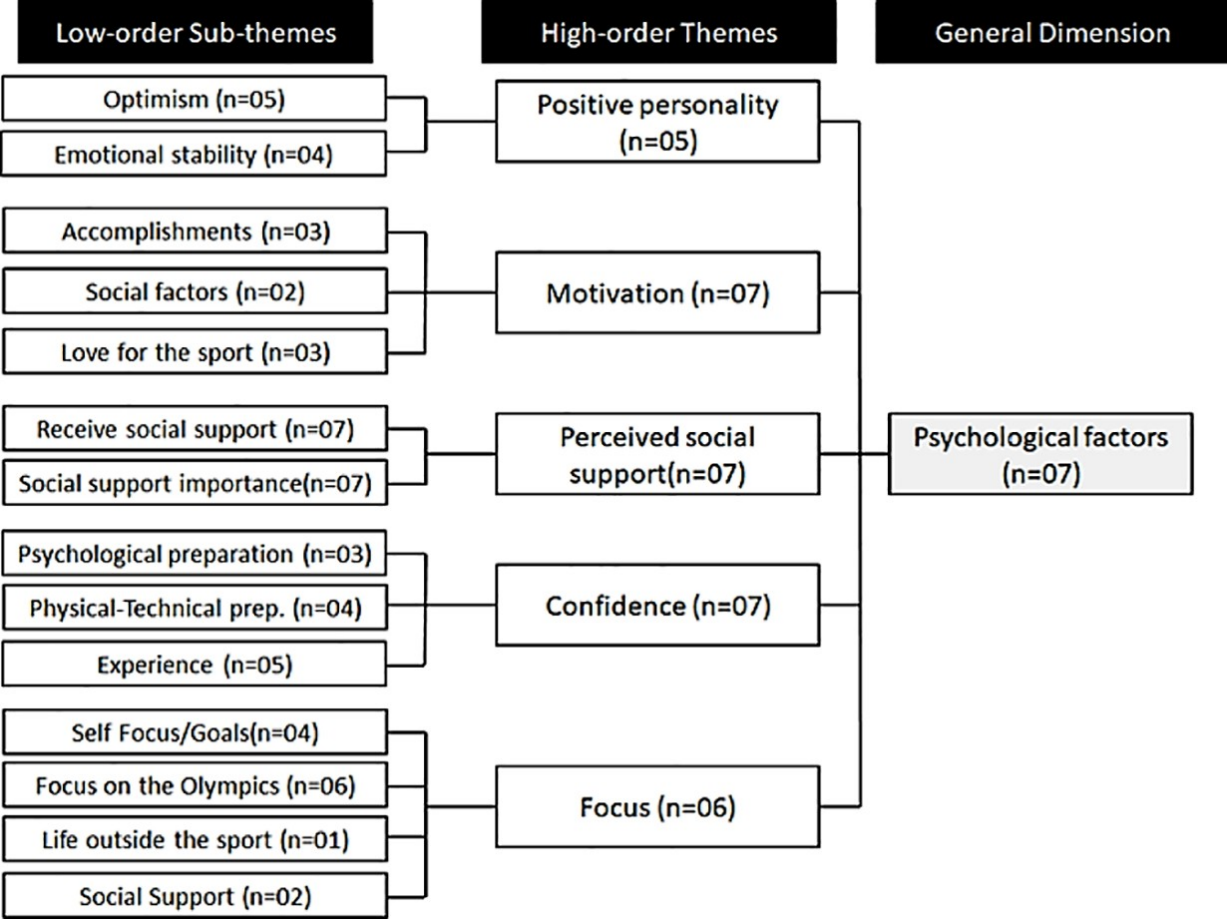
Psychological factors influencing the resilience process for the Brazilian rhythmic gymnastics athletes.

Through content analysis of the questionnaires, it was possible to identify the main stressors present in the elite rhythmic gymnastics context (Fig 3), athletes’ profile of appraisal and meta-cognition, psychological factors participating in the resilience process (Fig 4), facilitative responses presented by the athletes, as well as the athletes’ perception of performance. Among the main stressors faced by these athletes organizational stressors were the most prevalent, being mentioned by 06 of the 07 athletes (85.7%) showing their dissatisfaction toward the sport scoring/refereeing system, national sport policies in Brazil, and a supposed favoritism for certain countries in international competitions, as mentioned by Athlete 03: “… *the way that gymnastics is judged and politicking in championships with scores that often were not deserved by that country*, *but those are ‘superior’ teams in the sport*, *which end up taking a lead in the results*”.

Despite the 100% injury occurrence in the team, only two of the seven athletes mentioned injuries as being a stressful factor in their daily routines. On the other hand, five of them considered training to be a major source of adversities, mainly due to the physical and mental demands that affect the team as a whole: “*Physical and mental exhaustion in the day-to-day are huge*, *I think that these make training even harder sometimes*, *especially near competition*, *when training is not going as expected*” (Athlete 03). The least relevant stressor seemed to be the personal issues, highlighted by 03 athletes and without any emphasis on its negative impact, mainly saying that they avoid letting it affect their goals.

Analyzing the way that these stressors were seen and interpreted by the athletes, most of them (six) described aspects of their meta-cognition in their answers, showing their knowledge of their own emotions and thoughts, as well as positive ways of dealing with such. Four athletes mentioned having self-control over their emotions and behaviors, but only two answered that they face these stressors as challenges to be overcome, and only one athlete saw the context as a way of growth, saying that she deals “… *with sport as a personal challenge*. *A way to spiritually evolve*, *working on patience*, *persistence and self-control*” (Athlete 01). Despite athletes not necessarily seeing the adversities as negative, as referred to by Athlete 05, “*I am well resolved with these issues and it does not disturb me emotionally*,” the majority of them did not take such problems as challenges and ways to improve, showing a more neutral, low positive evaluation of stressors in this context.

When looking in to the psychological factors related to the athletes’ resilience process (Fig 4) it was observed that social support was the most important factor for facing adversities in the Brazilian elite rhythmic gymnastics context, mentioned by all athletes, who stated that they received support from family members and friends, besides attributing a high level of importance to it: “*Everyone gives me all the support I need*, *they send me positive notes and pray for me*. *Always staying in touch with them is already enough for me*. *They are always with me and this is very important for me*” (Athlete 05). It is worth mentioning that most of these athletes move from across the country to live far from their families in order to be part of the national team.

Another important aspect for the majority of these athletes was the focus on the 2016 Olympic Games, being the only common element for most of the athletes in the factors related to their focus. Moreover, four athletes highlighted the importance of being well prepared, both physically and psychologically, to reach their goals. Once again, social support appears in the answers of two athletes, stating that receiving social support is fundamental for them to stay focused on the sport.

When asked about the motivational factors that kept them in the elite context of the sport, intrinsic factors such as personal accomplishments and love for the sport were the most prevalent. However, extrinsic elements of motivation were also very relevant, for example their dreams for their futures, goals, and once again the social factor (family support), now as a motivational aspect for two of the seven athletes. To illustrate, Athlete 05 said: “*What motivates me is looking back at everything I’ve been through*, *seeing that today I am closer to fulfilling every athlete’s dream*,” referring to competing in the Olympic Games.

Despite the difficulties and the need for social support, most of the athletes (five) had shown to be optimistic, an important positive characteristic for the resilience process. Still, other elements of a positive personality, such as proactivity, innovation and extroversion, were not evident in their answers. Athletes had the use of past experiences as a common element for being confident. While trusting their psychological preparation was important for some of them, others mentioned their effort toward technical aspects, but in general all of them felt prepared for the competitions: “*I believe that we are well prepared*. *In my case*, *I try to be as rational as possible and not let the adrenaline get in my way*” (Athlete 06).

Facilitative responses are considered, in the model, to result from the interaction of stressors, one’s evaluation of such, and the set of psychological characteristics and variables involved. Six athletes addressed their facilitative responses and made evident that, although they try to think about their emotions, look for answers and think of ways to act, they did not mention an increased behavioral involvement or actual changes in decision-making toward their tasks. Three athletes stated that they get closer to their teammates in hard times, and only one stated she takes responsibility for her emotions, not letting them negatively affect the team. Thus, we noticed more emotional facilitative responses, with less evident behavioral responses.

Regarding athletes’ opinions about the ideal performance in rhythmic gymnastics, it was noticed that most of them (six out of seven) felt satisfied just with the possibility of participating in such important competitions, while three added some importance to their personal performance, referring to past victories as the most important moments in their careers. Moreover, two athletes mentioned that being able to train “happy and motivated” and to have an active social life and less stressful routines would be an ideal outcome, while three others valued the perfection, dedication toward training and a solid training basis since sport initiation as the ideal scenario.

Another investigated aspect was the sport injuries throughout these athlete’s careers. Despite mentioning some of the injuries that have occurred during their time in the sport, and three athletes complaining of severe injuries in their answers, the majority of them (six athletes) considered that they do not get injured frequently, and one athlete (Athlete 02), opposed to the others, stated that she mostly only goes through muscle soreness due to training: “*Thank god I am a person with a very strong body*, *it is very hard for me to get injured*, *only muscle soreness or even natural pain from training*”.

Five athletes described negative feelings toward injury occurrence, such as general negative feelings (*e*.*g*. “*I don’t feel well*”), loss of good mood, irritation and worry about getting worse or not getting better, as we can see in one of the athlete’s answer: “*I feel very bad when I get injured*, *I don’t like getting away from the sport*” (Athlete 05). On the other hand, four athletes have shown commitment to the rehabilitation process, mentioning focus, discipline and dedication toward some components of such process (rest, physical therapy, medication), besides a strong will to return to practice, with two athletes stating that they recover quickly.

I have been through some injuries, foot fracture, injury in the shoulder nerve and, the most recent, patellar luxation last year. Fortunately I always managed to recover before expected, having to stay little time away from training. I confess that in the beginning I get unmotivated, I can’t understand why this happens, but then this feeling turns into willpower, I dedicate a lot to physical therapy and recover myself fast. (Athlete 06)

**Fig 5:**
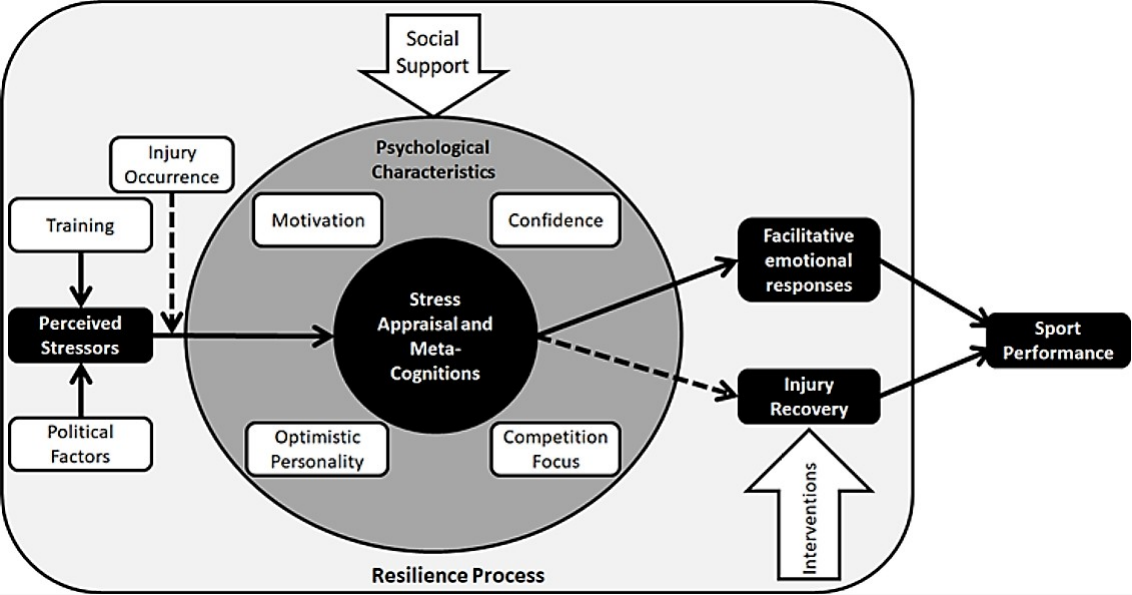
Hypothetical model of psychological resilience, injury and sport performance in the elite rhythmic gymnastics.

After analyzing the results, and based on the adopted theoretical framework for this investigation [3], a hypothetical model of the resilience process was developed for these athletes involved in Brazilian elite rhythmic gymnastics, considering its relationship to injuries and sport performance (Fig 5).

It was understood that these athletes perceived training, political refereeing issues and supposed unfairness in competitions as their main stressors. Through meta-cognitions, these athletes tried to deal with the negative feelings resulting from such adversities; in general, these adversities are not seen as challenges to be overcome or ways for growth, but are not necessarily seen as being barriers either.

These athletes’ resilience process is mainly based on social support provided by their loved ones, which is considered as an extremely important factor for them to stay in this context and being able to overcome eventual difficulties; besides social support, other intrinsic and extrinsic factors have shown to be relevant, such as love for sport, motivation for achievements, and the specific focus on the main competition, in this case, the Olympics; physical and psychological preparation, along with past experiences, make these athletes more confident. Moreover, their optimistic beliefs aid in this entire process. As a result of the interacting psychological characteristics, their appraisal, meta-cognitions, and the received social support, these athletes were able to interpret and control their emotions (emotional facilitative responses) when facing issues and adversities in the context of elite sports. However, the results did not provide elements to support a facilitated behavioral engagement.

Sport injuries also played a role in this model. Due to the way injuries were interpreted/described by the athletes, this factor was not included as a perceived stressor; however, it is still a relevant environmental stressor. The occurrence of an injury will be followed by a series of negative emotions which are natural but not desired. Such emotions and feelings will go through the center of the model, being influenced by its elements (perceived social support and optimism, for example). This process will help the athlete to overcome the initial negative emotions and more actively engage in rehabilitation. It is worth mentioning that external interventions, such as medication and physical therapy, will play an essential role for these athletes to recover, offering the necessary tools for them to exert their will to return to practice and directly participate in their own rehabilitation process. As an outcome, the athlete might present an adequate recovery, being able to return to sports-related activities and perform at an elite level.

## Discussion

The present investigation aimed to study the relations between resilience, stress and injury in the context of elite sports, more specifically, the Brazilian elite rhythmic gymnastics context. This investigation provides both quantitative and qualitative evidences for understanding of these three variables and their interaction, which is still a gap in sport psychology literature. In disagreement to our initial hypothesis, it was observed that regardless of the relatively controlled levels of stress and recovery, and athlete’s resilience levels, injury occurrence was still high and, thus, inevitable; moreover, such controlled levels of stress and recovery could not be directly linked to athletes’ resilience. However, psychological resilience seemed to play a role in the rehabilitation process, by potentially optimizing it and keeping the athlete involved and motivated toward the sport.

Broadening our understanding of the studied context and the results, it is noteworthy that the team had an adequate stress/recovery profile, with recovery levels being significantly higher than stress throughout the season, which is considered ideal for performance [25]. Athletes presented moderate-to-high levels of resilience, reflecting their ability to evaluate and deal with adversities, which was also supported by the team’s structure regarding psychological, medical and physical therapy interventions.

Results showed an absolute prevalence of injuries for all athletes, with consequent long periods of withdrawal from training and competition, even having three athletes dismissed from the team due to severe injuries that made competing, especially at the Olympic level, unfeasible. It is believed that injuries can cause the end of an athlete’s career [32], with evidences suggesting them to be the main reason for 20% to 47% of cases [33, 34]. A retrospective study by Ristolainen et al. [32] found that 9% of the sample (a total of 50 athletes) abandoned their sports, with 54% of them (27 athletes) reporting injury to be the main cause. In the present study, athlete number 07 presented the intention to quit her sport, as she describes her experience: “*I used to never get injured*, *but one day I got severely injured*, *I wanted to go back to the sport*, *did everything right to recover*, *took the medication and care of myself*, *but I left* [the training center] *and it seems like everything suddenly collapsed*. *I don’t feel like going back*”.

This one athlete represents 12.5% of the sample, and it is clear that the injury was the main cause leading this athlete to experience negative emotions and the intention to quit the sport. Moreover, this athlete had a substantial decrease in her resilience levels (-15.4%) during the study period. Based on this result, we could speculate that the frustration of an athlete’s personal strivings during the resilience process (which includes rehabilitation) might have negative and undesirable consequences, highlighting the importance of achieving positive results to offer a rewarding feedback to the athlete, supporting a successful resilient reintegration in the face of a stressor [35]. In that sense, the team and their staff have made efforts to obtain and acknowledge positive results when facing adversity. The work of a sport psychologist is essential in that matter, because even though positive results may be occurring, it is important that the athlete recognize them.

Despite this athlete’s case, positive relationships between resilience and the rehabilitation process were found, through qualitative analysis, for the other athletes. Due to the athletes’ perception of their environmental stressors, injuries were not necessarily considered as part of the main stressors, however, following injury occurrence, it is believed that these athletes, mostly influenced by social support, but also by their focus on the competition, motivation, love for the sport, confidence in their preparation/training and optimism, were able to overcome the resulting negative emotions and feelings, and then dedicated themselves toward overcoming their injury [3]. This includes mobilizing positive emotions and behaviors which favor the rehabilitation process, resulting in a faster return to training and even competition. Such assumptions are in agreement with evidences found in the literature, suggesting that resilient athletes possess attitudes and behaviors favorable to the rehabilitation process [1, 36–38].It also supports the Biopsychosocial Model of sport injury rehabilitation [39], which suggests a direct link between cognitive, affective and behavioral responses to the outcomes of rehabilitation.

A core element of the model proposed by Fletcher & Sarkar [3] is the stressors’ challenge appraisal, in the present study we evidenced low levels of such. Seeing a stressor as a challenge to be overcome is mostly a positive form of appraisal. Although the athletes did not necessarily see adversities in a negative way (as barriers, for example), they also would not see them as a challenge for which to improve themselves. Sport psychologists and other professionals working with athletes, not only on an elite level, should foster a positive way of interpreting the athletes’ adversities.

Considering the psychological factors influencing the resilience process [3], results have shown that social support was the most prevalent and most important variable for these athletes to be able to deal with the entirety of adversities that they face, as well as staying in the elite context. In this sense, social support was placed on a different level of importance in our hypothesized model, even preceding other factors. Attributing such high level of importance to social support seems to be a characteristic of female athletes [40], who consider the perception of social support in times of adversity as a factor of great importance for their resilient growth [4].

Besides social support, it was noticed that both intrinsic and extrinsic factors were necessary to produce facilitative responses. In our study, love for the sport, personal accomplishments, focus on the main competition, the goals established, and confidence in the physical and psychological training all worked together to keep these athletes motivated and engaged in their activities; however, the facilitative responses evidenced here were mostly emotional and not behavioral, differing from the original model [3]. Therefore, we highlight that both intrinsic and extrinsic sources of motivation should be proportional to the contextual demands in order to obtain success in training and competition. In this sense, the team reached the expected results for the two main competitions (Pan-American Games and Olympic Games), which was also possible due to the adequate levels of stress and recovery.

Adversities seem to have played an important and positive role in these athletes’ resilience development, which was evidenced through some level of increase in the resilience score of most of the athletes, as well as in their increased self-trust, use of past experiences, being motivated by their accomplishments and staying optimistic. A series of studies also show the sports context to be important for developing and strengthening psychological resilience [6, 41–46]. It seems that the context of sport can have all of the required elements for resilience growth; however, there should be enough adversities to challenge the athlete. Coaches, sport psychologists, families and everyone involved should promote positive resources to match the demands and allow the athlete to grow and to recognize such growth.

Despite the contributions, some limitations must be highlighted. First, opting for the elite level of this sport in Brazil meant having a very small sample, which limits the use of quantitative methods substantially; however, these athletes represent the elite in Brazilian rhythmic gymnastics, and adding qualitative data offered more elements to understand the results. Another limitation was the impossibility of directly interviewing the athletes due to the coach’s very strict policies. The alternative of using a structured questionnaire with open answers, though, provided enough elements for the investigation but we recognize that some information may have been missed due to this approach. Lastly, no physiological data, such as cortisol levels, were collected, which could also have provided more information about stress and recovery levels throughout the season, and possible relationships with psychological resilience.

We suggest that future investigations aiming to study the relationship between resilience, stress and injuries use a larger sample size, which may require the inclusion of different competitive levels and not only the elite, and also the use of other quantitative instruments to assess other correlated psychological variables. Studies with rhythmic gymnasts should further assess the structure and functionality of the model here presented, but always referring to the original “grounded theory of psychological resilience and optimal sport performance” [3] as well. In order to increase the overall understanding of the studied variables other sports should also be investigated. Assessing multiple variables in a large sample can also allow the use of more advanced and robust statistical methods, such as structured equation modelling.

## Conclusions

We conclude that psychological resilience can play a significant role in the process of injury rehabilitation, but not on injury occurrence. Resilient athletes will overcome the unpleasant feelings and emotions following an injury and better deal with the negative psychological aspects of it; these athletes can then engage more actively and positively toward rehabilitation, possibly speeding up their return to practice. It was not possible to establish direct relationships between stress and resilience, limiting the understanding of what extent psychological resilience can affect the undesirable consequences of stress that can be directly linked to injury (*e*.*g*. increased muscle tension, impaired flexibility and motor coordination), which is also suggested to be addressed by future investigations.

Professionals working with rhythmic gymnasts should have in mind the importance of social support for these athletes, not prohibiting their contact with supporters (*e*.*g*. family and friends) and might even include some level of psychological intervention or counseling with the main sources of social support for each athlete to insure that everyone involved is “speaking the same language”. Helping athletes to understand and deal with emotions, recognize their efforts and improvements, as well as promoting behavioral engagement and proactivity, are some key points to be addressed by the work of the sport psychologist.
